# Supplementary material for: Evaluating the quality of the 1000 genomes project data
Source: BMC Genomics. 2019 Aug 16;20:620. doi: 10.1186/s12864-019-5957-x (PMC6696682; doi:10.1186/s12864-019-5957-x)
Supplement: Supplementary file 1 — Figure S1. Representative distribution of INFO scores for chromosome 1 in HG00250 in (a) experimental SNPs (b) all 1000G SNPs. Figure S2. Imputation error in experimental SNPs after filtering low INFO score (< 0.3) SNPs (a) Total imputation error (b) imputation error as a function of minor allele frequency. Figure S3. Imputation error in all 1000G SNPs after filtering low INFO score (< 0.3) SNPs (a) Total imputation error (b) imputation error as a function of minor allele frequency. (DOCX 593 kb) [file 12864_2019_5957_MOESM1_ESM.docx]

# Supplementary Information for “Evaluating the quality of the 1000 Genomes Project data”

Figure S1: Representative distribution of INFO scores for chromosome 1 in HG00250 in (a) experimental SNPs (b) all 1000G SNPs

Figure S2: Imputation error in experimental SNPs after filtering low INFO score (<0.3) SNPs (a) Total imputation error (b) imputation error as a function of minor allele frequency

Figure S3: Imputation error in all 1000G SNPs after filtering low INFO score (<0.3) SNPs (a) Total imputation error (b) imputation error as a function of minor allele frequency


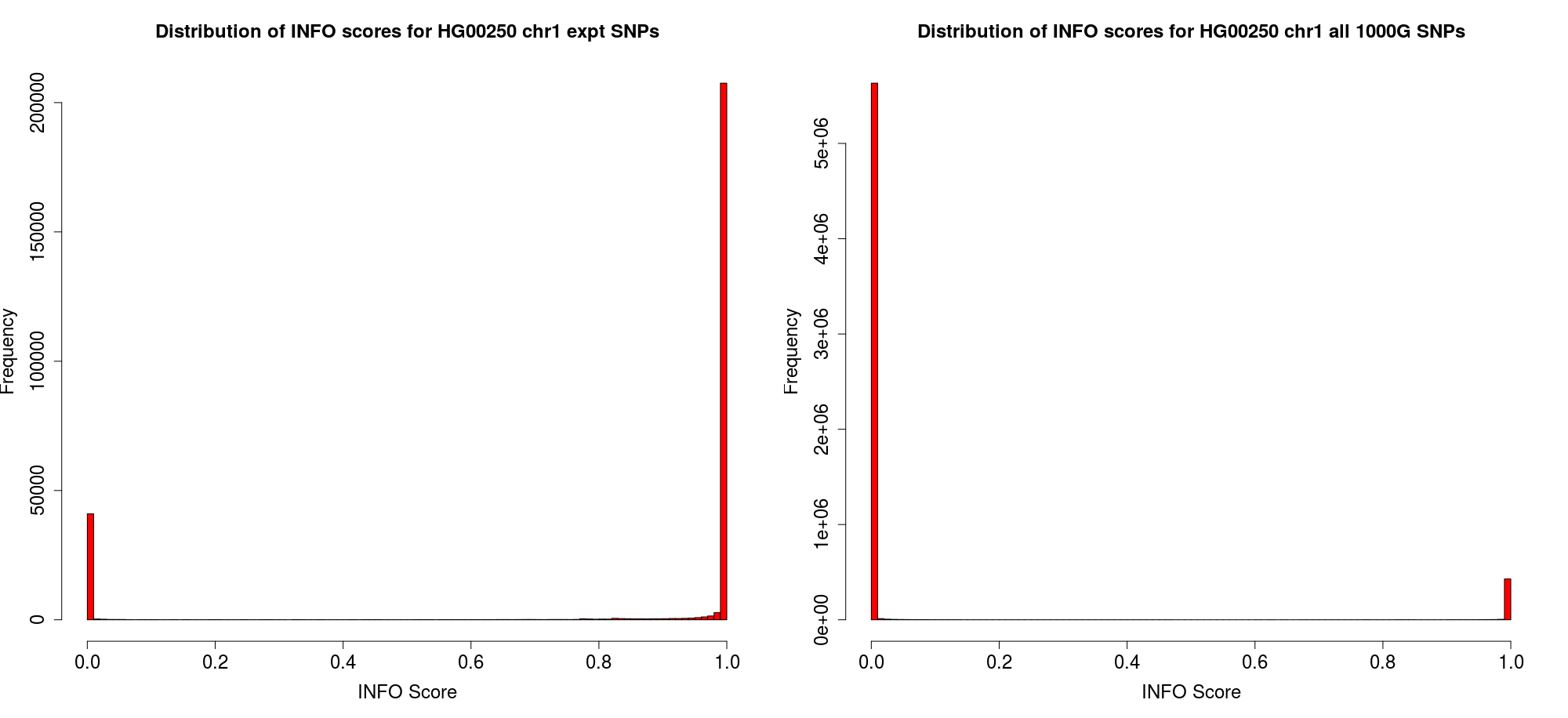


Figure S1


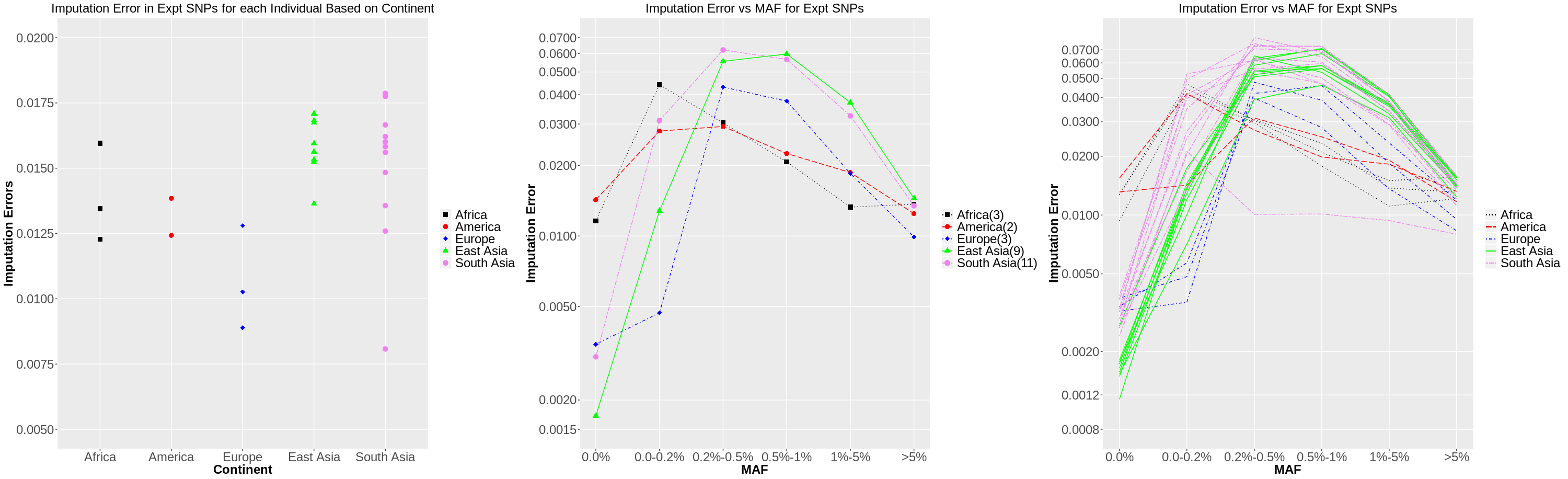


Figure S2


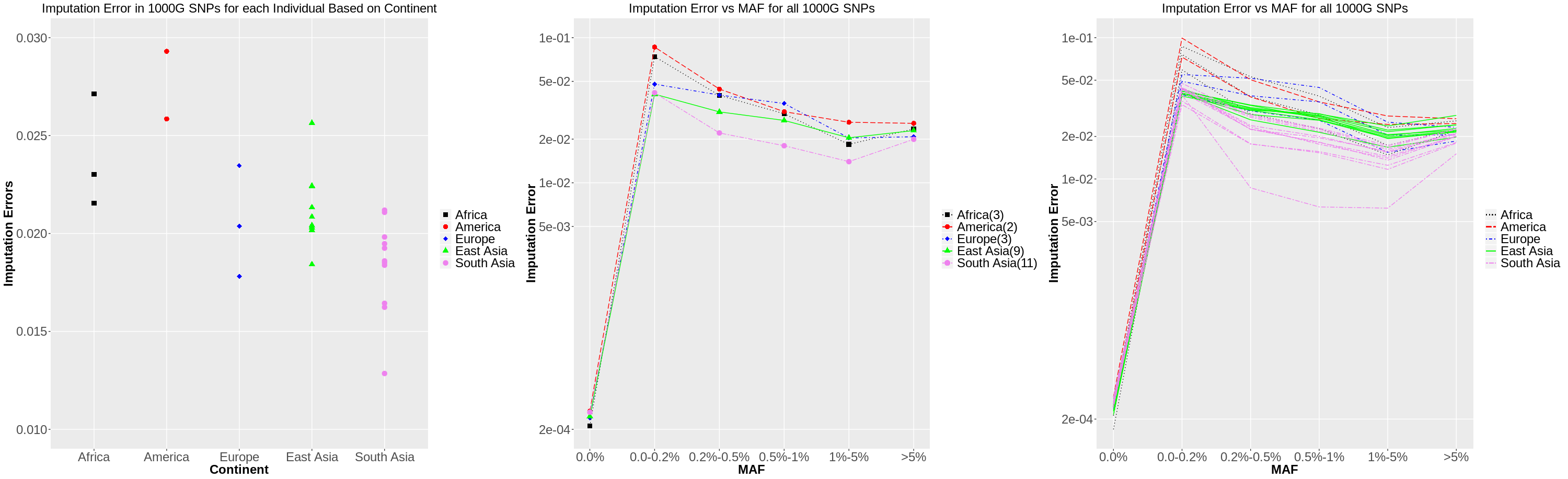


Figure S3
